# Supplementary material for: SAKE: Strobemer-assisted k-mer extraction
Source: PLoS One. 2023 Nov 29;18(11):e0294415. doi: 10.1371/journal.pone.0294415 (PMC10686461; doi:10.1371/journal.pone.0294415)
Supplement: Attachment — Submitted filename: Response to Reviewers.pdf [file pone.0294415.s001.pdf]

Dear Reviewers,

We thank you for your helpful comments. Below, we give our point-by-point responses the comments (skipping the parts with only Yes/No answers). The reviewers' comments are highlighted in yellow.

Reviewer #1: *It is a well-written and insightful piece of work that presents a valuable contribution to the field of bioinformatics. Your tool's approach to k-mer extraction is both innovative and promising, offering great potential for various applications.*

We thank the reviewer for their kind appraisal of our work.

Reviewer #2: *Leinonen and Salmela's manuscript "SAKE: Strobemer-assisted k-mer extraction" tackles the problem of determining the presence of **long** k-mers in the ~100-mer regime from a sequenced but unaligned error-prone long-reads (e.g. Oxford Nanopore data). They propose a modification to the Strobemer scheme of Sahlin and company, paired with a partial-order alignment, to allow for finding k-mers even in the presence of indel errors. Overall, the paper is well-motivated and a pleasure to read. Additionally, all code and data appear to be available.*

Thank you, we are glad to hear that the manuscript was enjoyable to read.

*Important note: although SAKE is compared to DSK and LoMeX, SAKE is most comparable to LoMeX (by the same pair of authors), which is also a k-mer extractor, but **not** a k-mer counter. On the other hand, the substantially more-used DSK counts k-mers, rather than simply establishing presence, providing strictly more information.*

This is indeed correct. SAKE does not provide k-mer counts so it is not meant to be used in applications that require strict counts (such as k-mer spectra analysis tasks based on k-mer counts). LoMeX estimates the counts and something similar could probably be done with SAKE. However, this was a lower-priority functionality that we decided to leave as future work if SAKE proved to be a practical tool on its own.

In terms of accuracy for long k-mers in the presence of indel error, SAKE is a clear winner over both DSK and LoMeX. DSK does not attempt to deal with errors, while LoMeX only handles substitution error, and this shows in the benchmarking. Unfortunately, this comes at a substantial cost in terms of time, as SAKE is also much slower. Indeed, the authors also ran a much-needed benchmark on corrected reads, where the advantage of SAKE more or less disappeared. From a practical point of view, at the present time, from a computational perspective, it's hard to make a clear decision of SAKE over correction+DSK, though I commend the authors on including that benchmark.

This was indeed quite unfortunate that SAKE ended up not being as fast as we had hoped. The initial idea was to implement it as a proof-of-concept program, and with the help of a more skilled programmer, it could probably be made faster. However, we are not sure if this is the correct direction to take. Maybe it would be better to use SAKE as an inspiration to come up with another more efficient technique to solve the problem.

I personally would've liked to see a benchmark comparing SAKE with assembly or mapping followed by k-mer extraction on the assemblies. One of the problems with framing the task as k-mer extraction instead of k-mer counting is that the results are a strict subset not just of the k-mer counting problem, but also the assembly problem, and SAKE thus has to compete with very well-optimized assemblers, for that is a major bioinformatics task. Looking at the runtimes, I think SAKE likely has a substantial edge on assembly, but not necessarily on mapping, and it would be useful to the end-reader to see that comparison. Personally, given the maturity of assemblers and mappers, I think that in most settings where I'd want long k-mer extraction, I'd probably just go with those tools instead, so it'd be helpful for the authors to comment specifically on more cases where k-mer extraction fulfills a bioinformatics need.

Since mapping tools require a reference genome and thus have additional information at their disposal as compared to SAKE we decided that doing such experiments would not necessarily be fair. However, the idea of first assembling the reads we found interesting, and decided to perform the experiments. As expected,

the assembly was quite slow (at least with the chosen assembler Canu). It seems to us that in the end, it might be just better to use a read correction tool instead.

Assembly in itself is one task where k-mer extraction can be needed. As an example, de Bruijn graph-based assembly has again gained popularity for assembling HiFi reads (see eg. the La Jolla assembler or MBG). These assemblers use large values of k. However, if HiFi reads are available, k-mer extraction becomes easier as the reads are very accurate and thus an approach like SAKE may not be needed in such cases.

Overall, I think what makes SAKE most interesting is in its adaptation of strobemers to compensate for indels in the reverse-complement setting of k-mer extraction. This technical contribution I could see taking on a life of its own outside of SAKE, and is I think sufficient even alone to merit a place in the published scientific record (as PLOS ONE describes in its reviewer guidelines). As such, SAKE as a whole certainly is a nice little tool which I could see being usefully applied in certain niche settings.

We appreciate the kind words. The reverse-complement variant of strobemers is indeed a novel idea we designed for grouping sequences regardless of orientation. We can see that this approach could be explored further to eventually find more use cases outside of SAKE.
